# Supplementary material for: Clinical profile of alcohol dependent paintents according to Lesch Typology one year after the Covid-19 pandemic-comparative study
Source: PLoS One. 2025 May 23;20(5):e0324851. doi: 10.1371/journal.pone.0324851 (PMC12101639; doi:10.1371/journal.pone.0324851)
Supplement: Attachment — Submitted filename: Completed.pdf [file pone.0324851.s002.pdf]

PLOS ONE

Clinical Profile Of Alcohol Dependent Paintents According To Lesch Typology One Year After The Covid-19 Pandemic-Comparative Study

--Manuscript Draft--

**Manuscript Number:**	PONE-D-24-35321R1
**Article Type:**	Research Article
**Full Title:**	Clinical Profile Of Alcohol Dependent Paintents According To Lesch Typology One Year After The Covid-19 Pandemic-Comparative Study
**Short Title:**	Clinical Profile Of Alcohol Dependent Paintents
**Corresponding Author:**	Dusan Petar Kuljancic, Ph.D.<br>University of Novi Sad Medical Faculty: Univerziteta u Novom Sadu Medicinski Fakultet<br>Novi Sad, Serbia SERBIA
**Keywords:**	alcoholism; COVID-19 pandemic; Lesch typology; vaccination, liver damage; neurological damage
**Abstract:**	Abstract: Background<br><br>The COVID-19 pandemics caused both physical and mental health problems raising global social tension, anxiety and discomfort which toghether lead to the increase in the consumption of psychoactive substances, among which alcohol was the most common, as a way of self-help. The hypothesis of this paper is the rising number of type II (anxious model) and type III (depressive model) alcohol dependent patients (as identified by the Lesch Typology) in the post-COVID-19 pandemic period compared to the pre-pandemic period, as a likely consequence of the stress, fear, problems and adversities that were caused by the pandemic.<br><br>Method<br><br>The research was conducted as a retrospective cross-sectional study. It included 218 patients who were diagnosed with alcohol dependence. To classify the patients by the Lesch Typology, the MS Windows softer package for data processing available in public domain was used.<br><br>Results<br><br>In relation to the Lesch Typology, 111 (50.9%) patients belonged to type III, 45 (20.6%) to type I, 37 (17.0%) to type II and 25 (11.5%) to type IV.<br><br>Conclusion<br><br>Compared to the pre-pandemic findings of alcohol dependents classification according to the Lesch Typology, there was no increase in types II and III after the COVID-19 pandemic.
**Order of Authors:**	Dusan Petar Kuljancic, Ph.D.
	Jelena Mile Amidzic, Dr
	Lazar Ljubotin, Dr
	Djendji Siladji, prof. dr
	Mina Cvjetkovic Bosnjak, prof. dr
	Vladimir Knezevic, prof. dr
	Dragana Ratkovic, prof. dr
	Vanja Bosic, Dr
	Vesna Vasic, mr
	Branislav Sakic, Dr
	Sanja Bjelan, Dr

Powered by Editorial Manager® and ProduXion Manager® from Aries Systems Corporation

	Minja Abazovic, Dr
	Masa Comic, Dr
	Predrag Savic, Dr
**Response to Reviewers:**	Dear Academic Editor and Reviewers,<br>First of all, I would like to express my gratitude for your positive assessment of my scientific work. We are aware that there are certain shortcomings in our article and we are grateful for your comments, which we hope have significantly contributed to improving the quality and scientific value of our article.<br>I will now present our responses to your objections and suggestions. I hope we were able to respond to your requests and thereby improve the quality of the work enough to be eligible for publication.<br>Respons to the reviewer 1 comments:<br>1.A section to the discussion focused on “Pandemic Healthcare Disruptions and the Rise of Type I Alcohol Dependence” has been added to the discusion chapter and the matter has been extesively explained citing new literature sources. I hope we met your request.<br>2.The methodology for how the effect of the Impact of Vaccination on Hospitalization Outcomes in our sample was measured has been explained in more detail in the Metodology section. I hope you will find it satissfactory.<br>3.A more detailed "Limitations and Future Directions" section has been added to the conclusion addresing both reviewer`s comments. I hope it will be enough.<br><br>Respons to The Journal Requirements:<br>1.I hope my revised manuscript now meets the PLOS ONE's style requirements, including those for file naming. I included the tamplates you have suggested.<br>2.There are ethical and legal restrictions on sharing a de-identified data set, because data contain potentially identifying or sensitive patient information taking into account that those are psychiatric patients with the addiction diagnosis. We are oblidged to protect the privacy of the patients participants because this matter is so sensitive and easiy can geopardise the patients privacy and exposure them to the public wich could be disasterous and is not according to the law. The Ethical Commitee of Clinical Centre of Vojvodina oblidged us to act like this. That is why we stated that the data will be shared upon the request to the corresponding authors. The request from the Ethical Commitee of our institution can be made on the phone No. +381214843484.<br>3.We stated in the availability form like this according to our´s Ethical commitee decision on our data sharing protocol. The data can be shared only on request to the corresponding authors.<br>4.The ethics statement appear only in the Methods section of our manuscript.<br>5.A separate caption for each figure has been added in the manuscript.<br>6.Review of the reference list has been done to ensure that it is complete and correct.<br>Dear Editor and reviewers, I hope that with these minimal changes that we have made in our scientific work and according to your requests, it has now been improved and meets the criteria for publication.<br>I hope for a soon and positive answer.<br>Sincerely,<br>Dr. Dusan Kuljancic
**Additional Information:**	
**Question**	**Response**
**Financial Disclosure**<br><br>Enter a financial disclosure statement that describes the sources of funding for the work included in this submission. Review the submission guidelines for detailed requirements. View published research articles from *PLOS ONE* for specific examples.	The author(s) received no specific funding for this work.

This statement is required for submission and **will appear in the published article** if the submission is accepted. Please make sure it is accurate.<br><br>**Funded studies**<br>Enter a statement with the following details:<br>• Initials of the authors who received each award<br>• Grant numbers awarded to each author<br>• The full name of each funder<br>• URL of each funder website<br>• Did the sponsors or funders play any role in the study design, data collection and analysis, decision to publish, or preparation of the manuscript?<br><br>Did you receive funding for this work?	
**Competing Interests**<br><br>Use the instructions below to enter a competing interest statement for this submission. On behalf of all authors, disclose any competing interests that could be perceived to bias this work—acknowledging all financial support and any other relevant financial or non-financial competing interests.<br><br>This statement is required for submission and **will appear in the published article** if the submission is accepted. Please make sure it is accurate and that any funding sources listed in your Funding Information later in the submission form are also declared in your Financial Disclosure statement.<br><br>View published research articles from *PLOS ONE* for specific examples.	The authors have declared that no competing interests exist.

Powered by Editorial Manager® and ProduXion Manager® from Aries Systems Corporation

**NO authors have competing interests**<br><br>Enter: *The authors have declared that no competing interests exist.*<br><br>**Authors with competing interests**<br><br>Enter competing interest details beginning with this statement:<br><br>*I have read the journal's policy and the authors of this manuscript have the following competing interests: [insert competing interests here]*<br><br>* typeset	
**Ethics Statement**<br><br>Enter an ethics statement for this submission. This statement is required if the study involved:<br><br>• Human participants<br>• Human specimens or tissue<br>• Vertebrate animals or cephalopods<br>• Vertebrate embryos or tissues<br>• Field research<br><br>Write "N/A" if the submission does not require an ethics statement.<br><br>General guidance is provided below. Consult the submission guidelines for detailed instructions. **Make sure that all information entered here is included in the Methods section of the manuscript.**	The paper was written in accordance to the Declaration of Helsinki on medical research conduct. The Ethical Board of the Clinical Centre of Vojvodina gave their approval for the conduct of the research on 1st December 2023, registered under number 00-281.

Powered by Editorial Manager® and ProduXion Manager® from Aries Systems Corporation

**Format for specific study types**<br><br>**Human Subject Research (involving human participants and/or tissue)**<br>• Give the name of the institutional review board or ethics committee that approved the study<br>• Include the approval number and/or a statement indicating approval of this research<br>• Indicate the form of consent obtained (written/oral) or the reason that consent was not obtained (e.g. the data were analyzed anonymously)<br><br>**Animal Research (involving vertebrate animals, embryos or tissues)**<br>• Provide the name of the Institutional Animal Care and Use Committee (IACUC) or other relevant ethics board that reviewed the study protocol, and indicate whether they approved this research or granted a formal waiver of ethical approval<br>• Include an approval number if one was obtained<br>• If the study involved *non-human primates*, add *additional details* about animal welfare and steps taken to ameliorate suffering<br>• If anesthesia, euthanasia, or any kind of animal sacrifice is part of the study, include briefly which substances and/or methods were applied<br><br>**Field Research**<br><br>Include the following details if this study involves the collection of plant, animal, or other materials from a natural setting:<br>• Field permit number<br>• Name of the institution or relevant body that granted permission	
**Data Availability**<br><br>Authors are required to make all data underlying the findings described fully available, without restriction, and from the time of publication. PLOS allows rare exceptions to address legal and ethical concerns. See the PLOS Data Policy and FAQ for detailed information.	No - some restrictions will apply

Powered by Editorial Manager® and ProduXion Manager® from Aries Systems Corporation

A Data Availability Statement describing where the data can be found is required at submission. Your answers to this question constitute the Data Availability Statement and **will be published in the article**, if accepted.<br><br>**Important:** Stating 'data available on request from the author' is not sufficient. If your data are only available upon request, select 'No' for the first question and explain your exceptional situation in the text box.<br><br>Do the authors confirm that all data underlying the findings described in their manuscript are fully available without restriction?	
**Describe where the data may be found in full sentences. If you are copying our sample text, replace any instances of XXX with the appropriate details.**<br><br>• If the data are **held or will be held in a public repository**, include URLs, accession numbers or DOIs. If this information will only be available after acceptance, indicate this by ticking the box below. For example: *All XXX files are available from the XXX database (accession number(s) XXX, XXX.*).<br>• If the data are all contained **within the manuscript and/or Supporting Information files**, enter the following: *All relevant data are within the manuscript and its Supporting Information files.*<br>• If neither of these applies but you are able to provide **details of access elsewhere**, with or without limitations, please do so. For example:<br><br>*Data cannot be shared publicly because of [XXX]. Data are available from the XXX Institutional Data Access / Ethics Committee (contact via XXX) for researchers who meet the criteria for access to confidential data.*<br><br>*The data underlying the results presented in the study are available from (include the name of the third party*	There are ethical and legal restrictions on sharing a de-identified data set, because data contain potentially identifying or sensitive patient information taking into account that those are psychiatric patients with the addiction diagnosis. We are oblidged to protect the privacy of the patients participants because this matter is so sensitive and easiy can geopardise the patients privacy and exposure them to the public wich could be disasterous and is not according to the law. The Ethical Commitee of Clinical Centre of Vojvodina oblidged us to act like this. That is why we stated that the data will be shared upon the request to the corresponding authors. The request from the Ethical Commitee of our institution can be made on the phone No. +381214843484 or on the email address doktorspecijalistamedicine@gmail.com.

and contact information or URL).<br>• This text is appropriate if the data are owned by a third party and authors do not have permission to share the data.<br><br>* typeset	
Additional data availability information:	Tick here if the URLs/accession numbers/DOIs will be available only after acceptance of the manuscript for publication so that we can ensure their inclusion before publication.

Manuscript Click here to access/download;Manuscript;main doc.docx

PLOS ONE

Clinical Profile Of Alcohol Dependent Paintents According To Lesch Typology One Year After The Covid-19 Pandemic-Comparative Study

Dusan Kuljancic [1,2,*¶], Jelena Amidzic [1*¶], Lazar Ljubotin [1¶], Djendji Siladji [1,2¶], Mina Cvjetkovic Bosnjak [1,2¶], Vladimir Knezevic [1,2¶], Dragana Ratkovic [1,2¶], Vanja Bosic [1,2&], Vesna Vasic [1,2&], Branislav Sakic [1,2&], Sanja Bjelan [1,2&], Minja Abazovic [1,2&], Masa Comic [1,2&], Predrag Savic [1,2&]

[1] Department of Psychiatry and Clinical Psychology, Medical Faculty, University of Novi Sad, Novi Sad, Vojovodina, Serbia

[2] Clinics of Psichiatry, University Clinical Centre of Vojvodina, Novi Sad, Vojovodina, Serbia

[#a]Current Address: Department of Psychiatry and Clinical Psychology, Medical Faculty, University of Novi Sad, Novi Sad, Vojovodina, Serbia

[#b]Current Address: Clinics of Psichiatry, University Clinical Centre of Vojvodina, Novi Sad, Vojovodina, Serbia

* Corresponding authors

E-mail: 1. dusan.kuljancic@mf.uns.ac.rs (DK)

2. 014841@mf.uns.ac.rs (JA)

[¶]These authors contributed equally to this work.

[&]These authors also contributed equally to this work.

Acknowledgments. On this occasion, the authors would like to wholeheartedly thank the employees of the Psychiatry Clinic of the University Clinical Center of Vojvodina in Novi Sad for their technical, moral and advisory support during the preparation of this work, as well as for the time and space allocated for the collection of data used in the preparation of this study.

Abstract

Background: The COVID-19 pandemics caused both physical and mental health problems raising global social tension, anxiety and discomfort which toghether lead to the increase in the consumption of psychoactive substances, among which alcohol was the most common, as a way of self-help. The hypothesis of this paper is the rising number of type II (anxious model) and type III (depressive model) alcohol dependent patients (as identified by the Lesch Typology) in the post-COVID-19 pandemic period compared to the pre-pandemic period, as a likely consequence of the stress, fear, problems and adversities that were caused by the pandemic. Method: The research was conducted as a retrospective cross-sectional study. It included 218 patients who were diagnosed with alcohol dependence. To classify the patients by the Lesch Typology, the MS Windows softer package for data processing available in public domain was used. Results: In relation to the Lesch Typology, 111 (50.9%) patients belonged to type III, 45 (20.6%) to type I, 37 (17.0%) to type II and 25 (11.5%) to type IV. Conclusion: Compared to the pre-pandemic findings of alcohol dependents classification according to the Lesch Typology, there was no increase in types II and III after the COVID-19 pandemic.

Keywords: alcoholism; COVID-19 pandemic; Lesch typology; vaccination, liver damage; neurological damage.

1. Introduction

The COVID-19 disease was caused by the new coronavirus SARS-CoV-2 (Severe Acute Respiratory Syndrome Coronavirus-2), discovered in late 2019 in China, where the infection first occurred. Soon after it developed its pandemic potential with more than 100 million people being infected and 2 million died worldwide. It ranges from asymptomatic to sever, even lethal multisystem forms [1, 2]. However, in addition to physical, the COVID-19 pandemic also impacted people's mental health. It contributed to the increase of global social tension, anxiety and discomfort. There was an increase in the consumption of psychoactive substances, among which alcohol was the most common, as a way of self-help [3]. Killgore et al. have conducted a research to determine the relation between the lockdown and social isolation during the pandemic and the increased frequency of alcohol intake. They asked 5,931 individuals to complete the Alcohol Use Disorders Identification Test (AUDIT). Over a six-month period, the consumption of alcohol and likely dependence increased on a monthly basis for the individuals that were under stay-at-home restrictions compared to those who were not [4]. Due to the nature of their illness, psychiatric patients represent a vulnerable social group, and as such their reaction to the COVID-19 pandemic resulted in the worsening of their psychopathological symptoms, among which anxiety and depression were the most frequently recorded ones [3].

Treatment for alcoholism is a complex, long process which aims at establishing abstinence, behavioural changes and adopting healthy lifestyle habits. The success of treatment depends on the applied methods: with a conventional approach

(pharmacotherapy and psychotherapy) the favourable outcome is achieved by one-fifth of patients, whereas with the systematic model by almost three-quarters of patients. However, there is no universal treatment plan which can be applied to all alcohol dependent patients. In order to plan a treatment, triage a patient and design an individual treatment plan, it is essential to have a systematised classification which can provide clear guidelines on different ways of treatment and optimal therapy goals [5].

The Lesch Typology is based on the data gathered from a longitudinal, prospective study. By observing the alcoholism development and progression in 436 patients, 4 types of alcohol dependent patients were identified based on the patients' drinking patterns and origin of alcohol craving:

I. the "allergy model" (craving caused by alcohol)

II. the "anxiety model" (craving caused by stress)

III. the "depressive model" (craving caused by mood)

IV. the "conditioning model" (craving caused by compulsion).

The Lesch Typology has proven to be exceptionally useful in everyday clinical practice, as it offers both the classification of alcohol dependent patients and additional information on therapeutic goals, as well as the most appropriate treatment approach to each subtype of patients [6].

In 2017, ~~hence~~ before the COVID-19 pandemic, Vejnovic et al. conducted a survey of 105 alcohol dependents at the Clinic for Psychiatry, Clinical Centre of Vojvodina. The patients were classified in one of the four types based on the Lesch Typology. The majority

of patients belonged to type III (60% of the respondents), then to type II (21.9%), type IV (10.5%) and to type I (7.6%) [7].

Our paper aims to compare the sociodemographic and clinical characteristics of the patients admitted to hospital for alcoholism treatment a year after the COVID-19 pandemic to the aforementioned results of the 2017 study.

The working hypothesis of this paper is the rising number of type II and type III alcohol dependent patients (as identified by the Lesch Typology) in the post-COVID-19 pandemic period compared to the pre-pandemic period, as a likely consequence of the stress, fear, problems and adversities that were caused by the pandemic, especially the change of routine, set patterns of living, fewer social contacts and constant uncertainty. Furthermore, we studied the protective effect of anti-COVID-19 vaccine on somatic complications in alcohol dependent patients and the length of their hospitalisation.

2. Materials and Methods

The research was conducted as a retrospective cross-sectional study at the Addictions Unit of the Clinic for Psychiatry, Clinical Centre of Vojvodina in Novi Sad. It included 218 patients aged over 18 years who were diagnosed with alcohol dependence (ICD-10: F10.2) according to the ICD-10 criteria and who were admitted for treatment in the period from

3rd October 2022 to 31 October 2023, that is to say, after the last wave of the COVID-19 pandemic in Serbia in August 2022 [8]. All the patients included in the research gave their written consent to treatment and thus participation in this research when admitted to hospital. The patients with comorbidities, either psychiatric or somatic, were not excluded from the research. Furthermore, this is a comparative study as its findings are compared to the findings of a similar study carried out at the Clinic for Psychiatry 6 years earlier, that is, before the pandemic.

The data was collected by viewing and reviewing electronic medical records using the Clinical Information System (CIS). The data mentioned were accessed for research purposes from 1st December 2023 till 21st December 2023 and none of the authors had access to information that could identify individual participants during or after data collection. The following sociodemographic characteristics were used: gender, age, marital status and occupation, while the clinical characteristics included: the time period of alcohol consumption, the number of hospitalisations due to alcoholism, the duration of the last hospitalisation, lesions of the liver and nervous system, other psychiatric diagnoses, heredity to alcoholism, whether the patient had a COVID-19 infection as well as their COVID-19 vaccination status.

To classify the patients by the Lesch Typology, the MS Windows softer package for data processing available in public domain was used (www.lat-online.at). The obtained data was exported to Microsoft Excel and statistically processed. The IBM SPSS 27.0

software was used for descriptive and analytical processing of the data. The results were presented in tabular and graphical forms.

In order to determine the importance of vaccination against COVID-19 infection in the group of alcoholics, the length of the last hospitalization was used as the main characteristic. Vaccinated and non-vaccinated alcoholics were compared regarding the length of the last hospitalization. The length of hospitalization was taken as a marker because it is comprehensive and implies that there were no serious complications during treatment and that the course of treatment and the outcome were favorable. Given that this is an observational study, the outcome of the hospital treatment was not detailed, but it was generated and presented only by the length of the treatment.

The paper was written in accordance to the Declaration of Helsinki on medical research conduct. The Ethical Board of the Clinical Centre of Vojvodina gave their approval for the conduct of the research on 1st December 2023, registered under number 00-281.

3. Results

The research included 218 patients, of which 180 (82.6%) were male, and 38 (17.4%) were female (Figure 1).

Figure 1. Gender distribution of patients

The average age of the patients was 51 (SD±11) years. The youngest patient was 20, and the oldest was 77 years old. Based on their age, the patients were divided into several groups. The young adults group included patients ages 18 to 34, the group of the

early middle-aged adults comprised patients ages 35 to 50, middle-aged patients were 51 to 65 years old, and older adults over age 66. In our study, there were 87 (39.9%) middle-aged patients, 86 (39.5%) early middle-aged patients, 26 (11.93%) older adults and 19 (8.71%) young adults. The results are presented in a tabular form (Table 1).

Table 1. Patients' age range

Age group	Number of patients (n)	Percent (%)
**Middle-aged adults (51-65)**	87	39.9
**Young middle-aged adults (35-50)**	86	39.5
**Older adults (≥66)**	26	11.93
**Young adults (18-34)**	19	8.71
**Total**	218	100

Of the total number of patients, 79 (36.2%) were married, 75 (34.4%) were single, 58 (26.6%) were divorced, and 6 (2.8%) have lost their spouse/partner (Figure 2).

Figure 2. Patients' marital status

In relation to work status, 97 (44.5%) patients were unemployed, 94 (43.1%) were employed, and 27 (12.4%) were retired (Figure 3).

Figure 3. Patients' work status

The length of the average period of using alcoholic drinks was 20 (SD±11) years. The shortest period was 6 months, and the longest 50 years. The average number of hospitalisations of alcohol dependent persons was 2 (SD±1). The avreage duration of the last hospitalisation due to alcohol use disorder was 7 days (SD±5). The shortest hospitalisation lasted 1 day, and the longest 23 days.

Of the total number of patients, 161 (73.9%) did not have a positive family history of alcoholism, and 57 (26.1%) did (Figure 4).

Figure 4. Psychiatric heredity disposition to alcoholism

In relation to the Lesch Typology, 111 (50.9%) patients belonged to type III, 45 (20.6%) to type I, 37 (17.0%) to type II and 25 (11.5%) to type IV. The results are presented in a tabular form (Table 2).

Table 2. The Lesch Typology

Type by the Lesch Typology	Nubmer of patients (n)	Percent (%)
III	111	50.9
I	45	20.6
II	37	17.0
IV	25	11.5
Total	218	100

Of the total number of patients, 125 (57.3%) did not have liver damage, and 93 (42.7%) did (Figure 5).

Figure 5. Presence of liver damage

Of the total number of patients, 138 (63.3%) did not have neurological damage, and 80 (36.7%) did (Figure 6).

Figure 6. Presence of neurological damage

In addition to alcohol dependence, 117 (53.7%) patients had a psychiatric comorbid condition and/or symptom, and 101 (46.3%) did not. Out of the total of 176 psychiatric comorbid conditions and symptoms, 69 (39.2%) suffered from depression, 31 (17.6%) suicidality, 28 (15.9%) personality disorder, 23 (13.1%) anxiety, 22 (12 .5%) psychiatric disorders due to the use of psychoactive substances other than alcohol (heroin, marijuana, opiates, cocaine, speed and ecstasy) and 3 (1.7%) psychotic disorders. The results are presented in a tabular form (Table 3).

Table 3. Psychiatric comorbid conditions and symptoms

Psychiatric diagnosis	Number (n)	Percent (%)
Depression	69	39.2
Suicidality	31	17.6
Personality disorder	28	15.9
Anxeity	23	13.1
Disorders due to the use of PS other than alcohol	22	12.5
Psychotic disorders	3	1.7
Total	176	100

146 (66.9%) patients had a COVID-19 infection, of which 79 (36.2%) were treated on an ambulatory basis, and 62 (30.7%) in hospital conditions. The other 72 patients (33.1%) did not suffer from COVID-19 infection. The results are shown graphically (Figure 7).

Figure 7. Presence of COVID-19 infection

In relation to the vaccination status against COVID-19 infection, 112 (51.4%) patients received the vaccine, and 106 (48.6%) did not (Figure 8).

Figure 8. Patients' status of vaccination against COVID-19 infection

The Mann-Whitney test shows that there is a statistically significant difference in the average length of the last hospitalisation in relation to the COVID-19 vaccination status ($p<0.05$).

4. Discussion

Soon after its initial outbreak in China, the COVID-19 disease was declared a global pandemic by the WHO in March 2020 [9]. Alongside its great impact on people's physical health, social functioning and economics, this public health issue has a significant effect on their mental wellbeing, as well as on the life quality of each individual [10]. Restrictive measures such as isolation and quarantine, aimed at suppressing this contagious disease are the key to preventing the transmission of the virus, but they represent a psychological burden for a population, disrupting personal, family and social functioning. The consequences of the pandemic are significant among vulnerable groups such as psychiatric patients, who are on the social margin even in normal circumstances [11].

The change in day-to-day routine and set patterns of living imposed by the pandemic caused a sense of fear and anxiety among vulnerable individuals, thus increasing incidences of stress-related disorders, but also causing exacerbation of existing psychiatric disorders. Strict epidemiological control measures, acting as social stressors, may lead to serious mental disorders such as depression and/or anxiety among previously healthy individuals or represent a huge burden for the mentally ill [11]. The appearance of fear, anxiety and depressive symptoms heightens the desire to consume psychoactive substances such as alcohol, as a form of self-help, especially during stressful periods [12].

In this study, a statistically significantly higher presence of alcohol dependence was observed in males. In the United States of America (USA), alcohol use disorder diagnosed more often in men (7%) than in women (4%) [13]. The incidence of alcohol dependence

during the pandemic was higher among males in Canada, India, Iran and the USA [14]. The average age of patients dependent on alcohol was 51 years, with almost equal representation of young middle-aged and middle aged adults. The National Epidemiologic Survey on Alcohol and Related Conditions (NESARC) and the National Survey on Drug Use and Health (NSDUH) have similar results related to the age ranges of persons dependent on alcohol. The prevalence of alcohol use disorders decreases with age. The risk of developing alcohol dependence was higher among the respondents aged 30 to 54 years compared to those who were 55 years or older [15]. White et al. pointed to the increase in number of alcohol-related deaths in 2020 compared to 2019, with the highest mortality rate in the age group 55 to 64 years [16].

In relation to marital status, the majority of patients are married, a slightly smaller number are single, while a quarter are divorced. Research conducted in June 2021 showed that there was a lower chance of getting married, that is, a higher chance of divorce among the population of alcohol dependents [17]. In relation to their work status, the largest number of patients are unemployed, a slightly smaller number are employed, and the fewest are retired. In their research on the use of psychoactive substances in Israel during the COVID-19 pandemic, Levy and colleagues concluded that the differences in work status were significant only among the respondents who used illegal drugs but not alcohol. The occurrence of equal representation of the employed and unemployed among the alcohol dependent persons is explained by the wide availability, popularity and social acceptability of alcohol beverages in Israel, which is also the case in Serbia [18].

On average, the number of hospitalisations of alcohol dependents is 2, and the average duration of the last hospitalisation is 7 days. Ahacic et al. have conducted a study which included 576 patients admitted to hospital for alcohol dependence. Nearly 50% of the patients had been hospitalised only once before, 34% three times or more, and 18% twice. Most patients were readmitted for treatment in the period between 2 and 4 years after the last hospitalisation [19]. Our study has found that almost three quarters of patients have a negative family history of alcohol dependence. Edenberg and colleagues point to the complexity of alcohol dependence as a genetic disease with variations in a large number of genes which increase the risk of the disease. Among the identified genes, the ones that stand out are ADH1B and ALDH2, which are included in the metabolism of alcohol and have the greatest impact on the manifestation of alcohol dependence. Twin studies in the USA and Europe suggest that the concordance for alcohol dependence ranges between 45% and 65% [20].

In relation to the Lesch Typology, 51% of the patients belonged to Type III, 21% to Type I, 17% to Type II and 11% to Type IV. In the period between 2006 and 2007, Penha Zago-Gomes and Nakamura-Palacios have conducted research on alcohol dependent patients at the Universidade Federal do Espírito Santo hospital. Of 170 patients, 21.2% were classified as Type I, 29.4% as Type II, 28.8% as Type III, and 20.6% as Type IV. They concluded that, although different types of alcoholics have different clinical presentations, the age at first intake of alcohol and the length of withdrawal did not differ among the types according to Lesch’s Typology [21]. Jakovljevic and colleagues compared clinical

characteristics of alcohol dependents in Serbia and Austria in the period between 2011 and 2012. The sample of Serbian population showed a very high rate of anxiety disorders (89.8% compared to 26.5%), which was explained by traumatic historical events in Serbs. Suicidal tendencies, not related to alcohol use or withdrawal syndrome, are significantly higher in the Austrian population sample (1.6% compared to 13.2%). There was no difference between the two samples in Type IV patients (20.5% compared to 20.6%). The sample of our population had significantly more Type III patients (42.5% compared to 33.8%) [22]. When compared to the results of classification of alcohol dependents according to the Lesch Typology obtained by Vejnovic et al. in 2017, it is evident that there was no increase in type II or type III after the COVID-19 pandemic, hence we reject the working hypothesis. Almost the same number of Type IV and a slightly higher number of Type I dependents is observed in our study. A study on reaction of the population of 4 different countries (Lebanon, Portugal, Italy and Serbia) to the COVID-19 pandemic indicated that the respondents from Serbia had greater resistance to psychological difficulties and stress that the COVID-19 pandemic imposed with its epidemiological measures than the other participating countries. The authors explain that the reason for this lies in specific sociocultural characteristics of Serbian population, such as previous experience with epidemics and the wars in Yugoslavia in the period between 1991 and 1999 [23]. We explain the increase of Type I, somatic and neurological comorbidities by the circumstances of the pandemic which led to difficulties in receiving medical assistance, non- treatment or inadequate treatment of non-COVID-19 diseases and

conditions, given that the entire healthcare system was focused on the current pandemic. The COVID-19 pandemic has made significant disruption in health service delivery all around the world [24]. The unavailability of health care for non-COVID patients during the COVID-19 pandemic was caused both by the burden on the health system by a large number of COVID patients and by the overload and saturation of health workers who were mostly reassigned from their regular tasks to work in the COVID centers [25]. Takinig all mentioned together, it is clear why the COVID-19 pandemics has disrupted both preventive and curative services for communicable and noncommunicable diseases [25-27]. Many of essential services, like internal and neurological check-ups, diagnostic procedures, have been delayed by the healthcare facilities, [28-30] patients were also unable to attend follow-ups and acute care visits due to the fear and anxiety they experienced during the pandemic waves [31].

It was difficult for all categories of non-COVID patients to obtain adequate health care during the pandemic, and especially for vulnerable social groups such as the alcoholics. The pandemic itself and the changes caused by it in the health system led to an increase in tension and uncertainty among the alcoholics, which contributed to a change in the way of drinking alcohol as a coping mechanisam, which usually ment an increase in the amount and frequency of drinking, and easily led to the appearance of alcohol-induced disorders such as delirium tremens or abstinence syndrome, which led to acute hospitalization [32]. These disorders are a main feature of the first type of alcohol addicts according to the Lesh typology. The restricted access to healthcare system during the

pandemic may have influenced the patterns of alcohol dependence leading to the increase in somatic complication of alcocholisam and explaining our finding- the increase of Lesh Type I addicts in our sample. This discovery sheds new light on how external stressors, such as the unavailability of adequate health care, can lead to a change in the pattern of alcohol consumption.

Nearly 60% of patients have no lesions of the liver, that is, there is no elevation of the liver enzymes. In the study conducted in Kentucky, 69% of alcohol dependents admitted for treatment had elevated biomarkers (aspartate aminotransferase and cytokeratin 18) for liver damage, with women having more pronounced lesions compared to men [33]. Neurological damage was present in 37% of patients. Meta-analysis results from 2018 indicated a prevalence of 46% of alcoholic dependents with peripheral neuropathy [34].

In addition to alcohol dependence, most patients suffered from a psychiatric comorbid condition or symptom. Depression was the most prevalent with nearly 40%, followed by suicidality, personality disorder, anxiety and the use of psychoactive substances other than alcohol with almost the same share, and there were only three patients with a psychotic disorder. Comorbid psychiatric disorders in patients with alcohol dependence are very common. On the one hand, alcohol dependence can induce the appearance of psychiatric disorders and on the other, disorders such as depression and anxiety can cause excessive intake of alcoholic beverages as a form of self-medication

[35]. Depressive disorders are most common among alcohol dependents, especially major depressive disorder with a prevalence of 33% as found in a 2019 study [36].

67% of patients were infected with COVID-19. The number of patients treated in an outpatient setting was almost equal to the number of those who were treated in hospitals. 52% of patients received a vaccine against COVID-19. Bailey et al. compared the course of the COVID-19 disease among patients with and without alcohol dependence. They proved that, when infected with SARS-CoV-2 virus, alcohol dependant patients have a higher chance of being admitted to hospital compared to patients without alcohol dependence. Moreover, the course of the disease is more sever, the complications are more prominent and mortality rates higher [37].

Literature data indicate the fact that patients with psychiatric disorders fall into a vulnerable category and if infected with the COVID-19 virus, they suffer more serious forms of the disease. In the population of psychiatric patients, the highest vaccination rate was among those with depression and anxiety disorders. On the other hand, patients suffering from psychotic disorders had the lowest rate of vaccination. There are several possible explanations as to why unvaccinated psychiatric patients had more severe forms of the COVID-19 infection. It is thought that they often ignored somatic symptoms and had less contact with primary health care. Also, it is possible that doctors focused more on the mental problems of these patients, and neglected somatic difficulties [38]. Findings from our study indicated a statistically significant difference in the average duration of the last hospitalisation compared to the vaccination status against COVID-19. The fully

vaccinated patients were hospitalised for alcoholism for a shorter period and the course of the treatment was without serious complications. Studies on COVID-19 vaccination in the period between January and May 2022 have shown high efficacy of all available vaccines in the period [39]. The vaccination led to a smaller number of critically ill patients requiring treatment in intensive care units and a lower mortality rate [40].

There are some deficiencies and possible limitations of this study. First, the design of the research, it self, as an observational study of a retrospective nature does not provide the ability to determin the causality between the examined phenomena. To overcome this problem and to better understand casual relationships, a longitudinal study is needed to validate our results and make them generalizable. Second, the sample size may not be sufficient to account for the sample representativness. It is necessary to conduct a similar study in cooperation with several different psychiatric institutions throughout Serbia and region to shed a new light on the the complexities of alcohol abuse during crises and draw validated conclusions.

5. Conclusions

Compared to the pre-pandemic findings of alcohol dependents classification according to the Lesch Typology, there was no increase in types II and III after the COVID-19 pandemic. This might be explained by specific sociocultural characteristics of Serbian population and their higher tolerance of difficulties and stress imposed by the pandemic.

However, we explain that the rise of type I alcohol dependents, with somatic and neurological comorbidities, was caused by the circumstances of the pandemic that led to difficulties in healthcare delivery and poor treatment of non-COVID related illnesses and the reorientation of the healthcare system to the pandemic treatment. More than a half of patients in our study had COVID-19, half of whom had been vaccinated. We have proven a positive effect of vaccination on the length and course of hospitalisation, which indicates the importance of implementing a full and comprehensive vaccination programme for vulnerable psychiatric patients, who are often neglected due to stigma.

Supplementary Materials: None.

Author Contributions: Conceptualization, D.K. and J.A.; data curation, L.L. and D.S.; formal analysis, D.K., V.V. and M.C.; investigation, V.K. and V.B.; methodology, B.S., J.A. and M.A..; project administration, S.B. and D.R.; resources, D.R. and D.S.; software, S.B. and V.B..; supervision, V.K. and B.S.; validation, V.V. and M.C.B.; visualization, L.L. and P.S.; writing—original draft, M.C. and M.C.B.; writing—review and editing, M.A. and P.S. All authors have read and agreed to the published version of the manuscript.

Funding: This research received no external funding.

Informed Consent Statement: Written informed consent was obtained from all subjects involved in the study.

Data Availability Statement: The data that support the findings of this study are available from the corresponding authors (D.K. and J.A.) upon reasonable request.

Conflicts of Interest: The authors declare no conflicts of interest.

References

1. Singhal T. A Review of Coronavirus Disease-2019 (COVID-19). Indian J Ped. 2020;87(4):281-6.
2. Teo I, Chay J, Cheung YB, Sung SC, Tewani KG, Yeo LF et al. Healthcare worker stress, anxiety and burnout during the COVID-19 pandemic in Singapore: A 6-month multi-centre prospective study. PLoS One. 2021;22;16(10):e0258866.
3. Kuljančić D, Cvjetković Bošnjak M, Vejnović AM, Bjelan S. Pandemija SARS-CoV-2 virusa i njen uticaj na mentalno zdravlje psihijatrijskih pacijenata u odnosu na psihički zdrave osobe. Timočki medicinski glasnik. 2021;46(4):161-8.
4. Killgore WDS, Cloonan SA, Taylor EC, Lucas DA, Dailey NS. Alcohol dependence during COVID-19 lockdowns. Psychiatry Res. 2021;296:113676.
5. Patel AK, Balasanova AA. Treatment of Alcohol Use Disorder. JAMA. 2021;325(6):596.
6. Schlaff G, Walter H, Lesch OM. The Lesch alcoholism typology - psychiatric and psychosocial treatment approaches. Ann Gastroenterol. 2011;24(2):89-97.
7. Vejnovic AM, Dickov A, Kneževic V, Ratković D, Kolundžija K, Ivetić O. Karakteristike osoba zavisnih od alkohola u odnosu na Lesh-ovu tipologiju. U: Burgić Radmanović M, Račetović G, urednici. IV Kongres psihijatara BiH; 2017 Nov 16-19; Banja Luka, BiH; p. 210.
8. Batut.org.rs [homepage on the Internet]. Godišnji izveštaj o zaraznim bolestima u Republici Srbiji za 2022. godinu. 2023 [updated 2023 August 19; cited 2024 February 13]. Available from: https://www.batut.org.rs/index.php?content=2671.

9. WHO Director-General’s opening remarks at the media briefing on COVID-19 - 11 March 2020 [Internet]. Available from: https://www.who.int/director- general/ speeches/detail/who-director-general-s-opening-remarks-atthe-media-briefing-on-covid- 19 11-march-2020.
10. Marković I, Nikolovski S, Milojević S, Živković D, Knežević S, Mitrović A et al. Uticaj poverenja javnosti i medija na nivoe anksioznosti i depresije među stručnim radnicima tokom COVID-19 epidemije u Srbiji. Vojnosanit Pregl. 2020;77(11):1201-9.
11. Kuljancic D, Cvjetkovic Bosnjak M, Siladji D, Hinic D, Veskovic D, Janjic N et al. The Role of COVID-19-Associated Fear, Stress and Level of Social Support in Development of Suicidality in Patients Diagnosed with Affective and Stress-Induced Psychiatric Disorders during the COVID-19 Pandemic-A Comparative Analysis. Brain Sci. 2023;13(5):812.
12. Roberts A, Rogers J, Mason R, Siriwardena AN, Hogue T, Whitley GA et al. Alcohol and other substance use during the COVID-19 pandemic: A systematic review. Drug Alcohol Depend. 2021;229(Pt A):109150.
13. White AM. Gender Differences in the Epidemiology of Alcohol Use and Related Harms in the United States. Alcohol Res. 2020;40(2):01.
14. Sarfraz A, Sarfraz Z, Sarfraz M, Thevuthasan S, Khan T, Inam I et al. The Incidence of Alcohol Use Disorders during the COVID-19 Pandemic. Psychiatr Danub. 2022;34(3):535-43.
15. Delker E, Brown Q, Hasin DS. Alcohol Consumption in Demographic Subpopulations: An Epidemiologic Overview. Alcohol Res. 2016;38(1):7-15.
16. White AM, Castle IP, Powell PA, Hingson RW, Koob GF. Alcohol-Related Deaths During the COVID-19 Pandemic. JAMA. 2022;327(17):1704-6.

17. Thomas NS, Kuo SI, Aliev F, McCutcheon VV, Meyers JM, Chan G et al. Alcohol use disorder, psychiatric comorbidities, marriage and divorce in a high-risk sample. Psychol Addict Behav. 2022;36(4):364-74.
18. Levy I, Cohen-Louck K, Bonny-Noach H. Gender, employment, and continuous pandemic as predictors of alcohol and drug consumption during the COVID-19. Drug Alcohol Depend. 2021;228:109029.
19. Ahacic K, Kennison RF, Kåreholt I. Alcohol abstinence, non-hazardous use and hazardous use a decade after alcohol-related hospitalization: registry data linked to population-based representative postal surveys. BMC Public Health 2014;14:874.
20. Edenberg HJ, Foroud T. Genetics and alcoholism. Nat Rev Gastroenterol Hepatol. 2013; 10(8): 487-94.
21. Penha Zago-Gomes M , Miyuki Nakamura-Palacios E. Tipologia de Lesch em alcoolistas no Brasil. J Bras Psiquiatr. 2009;58(3):169-74.
22. Jakovljevic M, Riegler A, Jovanovic M, Djordjevic N, Patek K, Lesch O et al. Serbian and Austrian Alcohol-Dependent Patients: A Comparison of Two Samples Regarding Therapeutically Relevant Clinical Features. Alcohol and Alcoholism. 2013;48(4):505- 8.
23. Öcal A, Cvetković V, Baytiyeh H, Santos Tedim FM, Zečević M. Public reactions to the disaster COVID-19: a comparative study in Italy, Lebanon, Portugal and Serbia. Geomatics, natural hazards and risk. 2020;11(1):1864-85.
24. Menendez C, Gonzalez R, Donnay F, Leke RGF. Avoiding indirect effects of COVID-19 on maternal and child health. Lancet Glob Health. 2020;8:e863–e864. doi: 10.1016/S2214-109X(20)30239-4.
25. Haileamlak A. The impact of COVID-19 on health and health systems. Ethiop J Health Sci. 2021 Nov;31(6):1073-1074. doi: 10.4314/ejhs.v31i6.1. PMID: 35392335; PMCID: PMC8968362.

26. WHO, author. Attacks on health care in the context of COVID-19. Geneva: WHO; [Jully 20, 2024]. https://www.who.int/newsroom/feature-stories/detail/attacks-on-healthcare-in-the-context-of-covid-19 .
27. WHO, author. COVID-19 significantly impacts health services for noncommunicable diseases. Geneva: WHO; [Jully 20, 2024]. https://www.who.int/newsroom/detail/01-06-2020-covid-19-significantly-impacts-health-services-for-noncommunicable-diseases .
28. Papautsky EL, Hamlish T. Patient-reported treatment delays in breast cancer care during the COVID-19 pandemic. Breast Cancer Res Trea. 2020;184(1):249–254. doi: 10.1007/s10549-020-05828-7.
29. Panagiotidis E. Nuclear medicine and oncology in the COVID-19 pandemic era. Hell J Nucl Med. 2020;23(Suppl):35–40.
30. Park C, Sugand K, Nathwani D, et al. Impact of the COVID-19 pandemic on orthopedic trauma workload in a London level 1 trauma center: the “golden month”. Acta Orthop. 2020;91(5):556–561. doi: 10.1080/17453674.2020.1783621.
31. Passos L, Prazeres F. Impact on mental health due to COVID-19 pandemic: cross-sectional study in Portugal and Brazil. Int J Environ Res Public Health. 2020;17(18):6794. doi: 10.3390/ijerph17186794.
32. Shuey B, Halbisen A, Lakoma M, Zhang F, Argetsinger S, Williams EC, et al. High-Acuity Alcohol-Related Complications During the COVID-19 Pandemic. JAMA Health Forum. 2024;5(4):e240501. doi:10.1001/jamahealthforum.2024.0501
33. Kirpich IA, McClain CJ, Vatsalya V, Schwandt M, Phillips M, Falkner KC et al. Liver Injury and Endotoxemia in Male and Female Alcohol-Dependent Individuals 19 Admitted to an Alcohol Treatment Program. Alcohol Clin Exp Res. 2017;41(4):747- 57.

34. Julian T, Glascow N, Syeed R, Zis P. Alcohol-related peripheral neuropathy: a systematic review and meta-analysis. Journal of Neurology. 2018;266:2907–19.

35. Dervaux A, Laqueille X. Comorbidités psychiatriques de l'alcoolodépendance. Presse Med. 2018;47(6):575-85.

36. McHugh RK, Weiss RD. Alcohol Use Disorder and Depressive Disorders. Alcohol Res. 2019;40(1):arcr.v40.1.01.

37. Bailey KL, Sayles H, Campbel J, Khalid N, Anglim M, Ponce J et al. COVID-19 patients with documented alcohol use disorder or alcohol-related complications are more likely to be hospitalized and have higher all-cause mortality. Alcohol Clin Exp Res. 2022;46:1023–35.

38. Bosnjak MC, Kuljancic D, Vejnovic AM, Hinic D, Knezevic V, Ratkovic D, et al. Rates of Vaccination against COVID-19 in Psychiatric Outpatients. J. Pers. Med. 2024;14(7):748.

39. Chirico F, Teixeira da Silva JA, Tsigaris P, Sharun K. Safety & effectiveness of COVID-19 vaccines: A narrative review. Indian J Med Res. 2022 Jan;155(1):91-104.

40. Gholinataj Jelodar M, Mirzaei S, Saghafi F, Rafieian S, Rezaei S, Saatchi A, Dehghani Avare Z, Dehghan Niri M. Impact of vaccination status on clinical outcomes of hospitalized COVID-19 patients. BMC Infect Dis. 2024 Feb 23;24(1):254.

Click here to access/download;Figure;Figure 1. Gender distribution of patients.docx

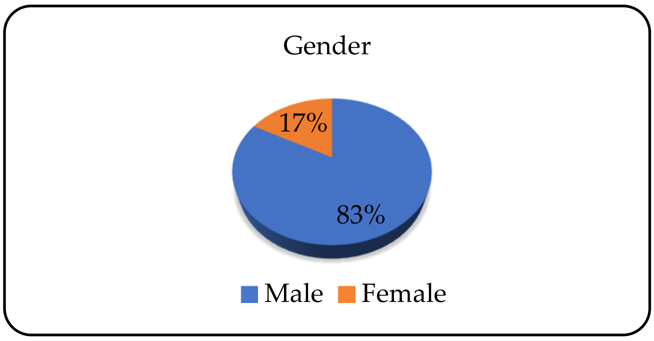

 Click here to access/download;Figure;renamed_03644.docx

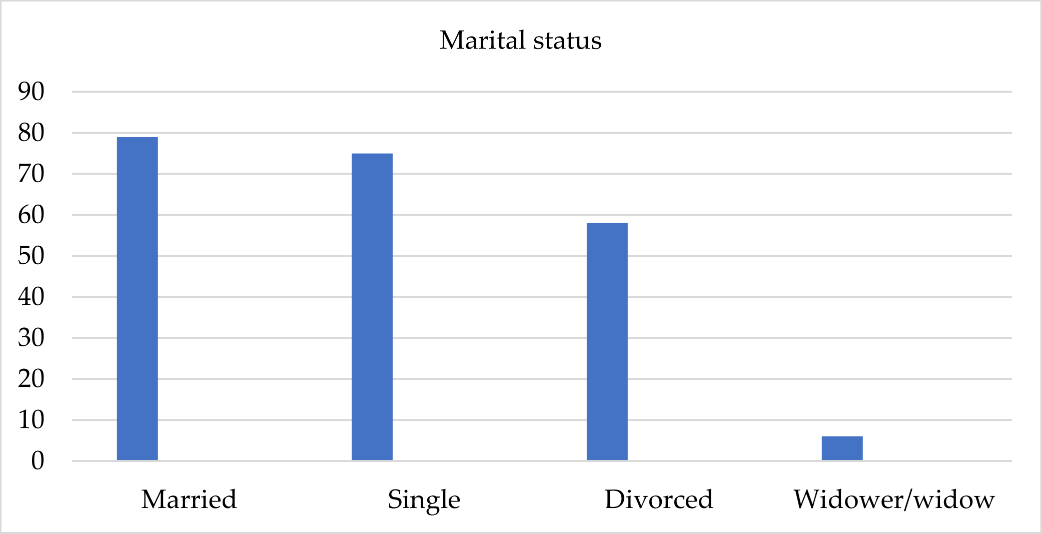

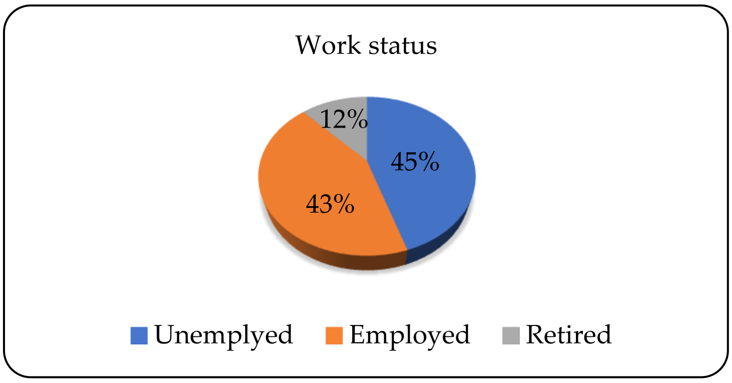
Work status
12%
45%
43%
Unemplyed
Employed
Retired

Click here to access/download;Figure;Figure 4. Psychiatric heredity disposition to alcoholism.docx

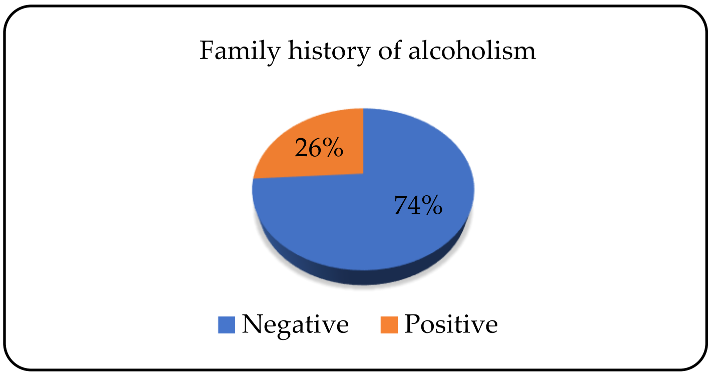

Click here to access/download;Figure;Figure 5. Presence of liver damage.docx

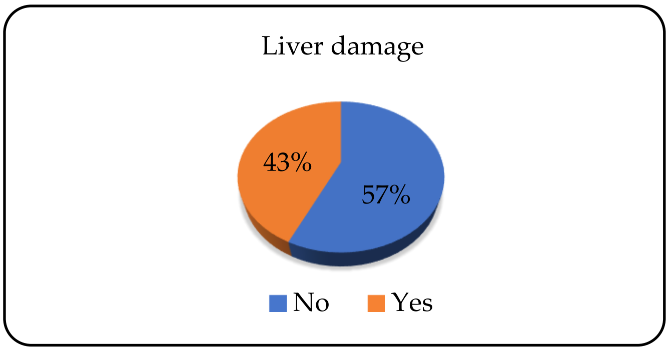

Click here to access/download;Figure;Figure 6. Presence of neurological damage.docx

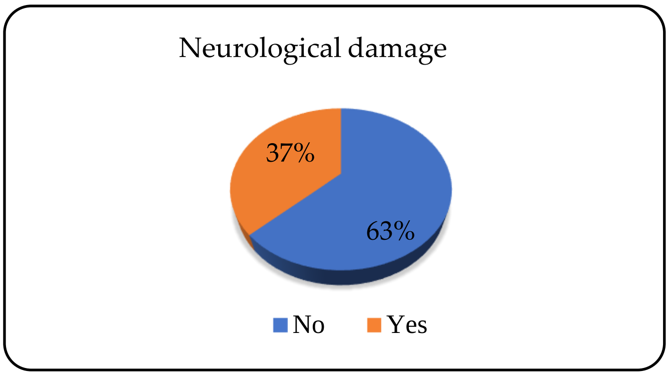

Click here to access/download;Figure;Figure 7. Presence of COVID-19 infection.docx

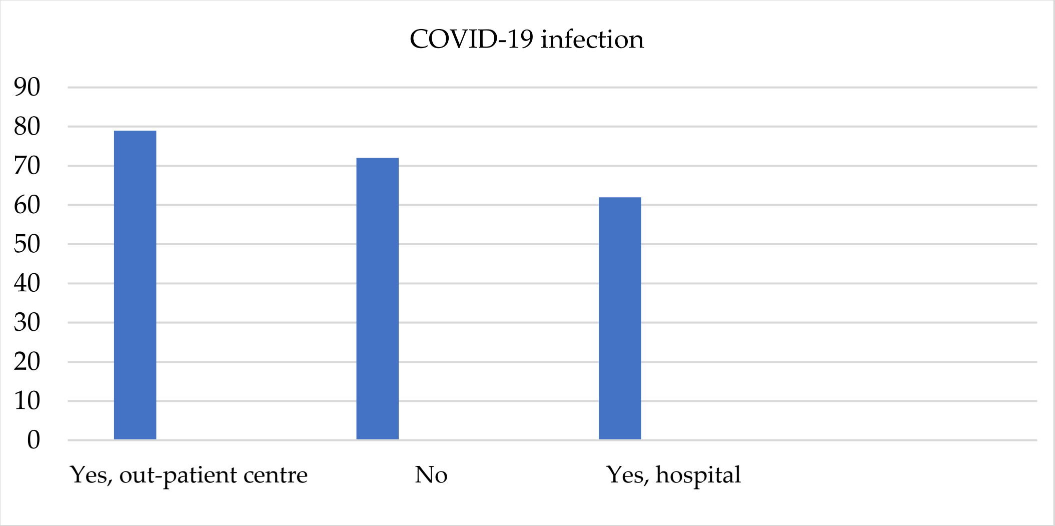

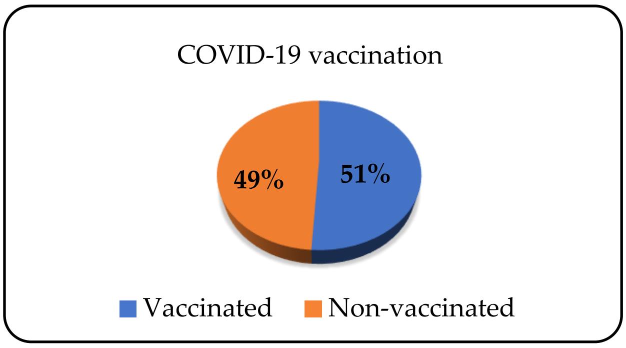
COVID-19 vaccination
49%
51%
Vaccinated
Non-vaccinated

Revised Manuscript with Track Changes

PLOS ONE

Clinical Profile Of Alcohol Dependent Paintents According To Lesch Typology One Year After The Covid-19 Pandemic-Comparative Study

Dusan Kuljancic [1,2,*¶], Jelena Amidzic [1*¶], Lazar Ljubotin [1¶], Djendji Siladji [1,2¶], Mina Cvjetkovic Bosnjak [1,2¶], Vladimir Knezevic [1,2¶], Dragana Ratkovic [1,2¶], Vanja Bosic [1,2&], Vesna Vasic [1,2&], Branislav Sakic [1,2&], Sanja Bjelan [1,2&], Minja Abazovic [1,2&], Masa Comic [1,2&], Predrag Savic [1,2&]

[1] Department of Psychiatry and Clinical Psychology, Medical Faculty, University of Novi Sad, Novi Sad, Vojovodina, Serbia

[2] Clinics of Psichiatry, University Clinical Centre of Vojvodina, Novi Sad, Vojovodina, Serbia

[#a]Current Address: Department of Psychiatry and Clinical Psychology, Medical Faculty, University of Novi Sad, Novi Sad, Vojovodina, Serbia

[#b]Current Address: Clinics of Psichiatry, University Clinical Centre of Vojvodina, Novi Sad, Vojovodina, Serbia

* Corresponding authors

E-mail: 1. dusan.kuljancic@mf.uns.ac.rs (DK)

2. 014841@mf.uns.ac.rs (JA)

[¶]These authors contributed equally to this work.

[&]These authors also contributed equally to this work.

Acknowledgments. On this occasion, the authors would like to wholeheartedly thank the employees of the Psychiatry Clinic of the University Clinical Center of Vojvodina in Novi Sad for their technical, moral and advisory support during the preparation of this work, as well as for the time and space allocated for the collection of data used in the preparation of this study.

Formatted: Font: Times New Roman, 10 pt

Formatted: Indent: Left: -0.12"

Formatted: Indent: Left: 0"

Formatted: Indent: Left: 0.15", Tab stops: 0.43", Left

Formatted: Font: Times New Roman, 12 pt

Formatted: Indent: Left: 0"

~~*Research Article*~~

~~Clinical Profile Of Alcohol Dependent Paintents According To Lesch Typology One Year After The Covid-19 Pandemic-Comparative Study~~

~~**Dusan Kuljancic [1,2,*], Jelena Amidzic [1*], Lazar Ljubotin [1], Djendji Siladji [1,2], Mina Cvjetkovic Bosnjak [1,2], Vladimir Knezevic [1,2], Dragana Ratkovic [1,2], Vanja Bosic [1,2], Vesna Vasic [1,2], Branislav Sakic [1,2], Sanja Bjelan [1,2], Minja Abazovic [1,2], Masa Comic [1,2], Predrag Savic [1,2]**~~

~~**1 University of Novi Sad, Medical Faculty, Hajduk Vejkova 3; 21000 Novi Sad;**~~

~~**2 University Clinical Centre of Vojvodina, Clinics of Psichiatry, Hajduk Veljkova 4; 21000 Novi Sad**~~

~~*** Correspondence: Dusan Kuljancic: dusan.kuljancic@mf.uns.ac.rs; +38125726287 and Jelena Amidzic: 014841@mf.uns.ac.rs; +381649944572; .**~~

Abstract~~:~~

Background: The COVID-19 pandemics caused both physical and mental health problems raising global social tension, anxiety and discomfort which toghether lead to the increase in the consumption of psychoactive substances, among which alcohol was the most common, as a way of

Formatted: Font: Times New Roman, 18 pt, Bold

Formatted: Heading 1, Indent: Left: 0", First line: 0", Line spacing: single

Formatted: Font: (Default) Times New Roman, 18 pt

Formatted: Heading 1, Indent: Left: 0", Space Before: 0 pt, Line spacing: single

Formatted: Font: Not Bold

self-help. The hypothesis of this paper is the rising number of type II (anxious model) and type III (depressive model) alcohol dependent patients (as identified by the Lesch Typology) in the post-COVID-19 pandemic period compared to the pre-pandemic period, as a likely consequence of the stress, fear, problems and adversities that were caused by the pandemic. Method: The research was conducted as a retrospective cross-sectional study. It included 218 patients who were diagnosed with alcohol dependence. To classify the patients by the Lesch Typology, the MS Windows softer package for data processing available in public domain was used. Results: In relation to the Lesch Typology, 111 (50.9%) patients belonged to type III, 45 (20.6%) to type I, 37 (17.0%) to type II and 25 (11.5%) to type IV. Conclusion: Compared to the pre-pandemic findings of alcohol dependents classification according to the Lesch Typology, there was no increase in types II and III after the COVID-19 pandemic.

Keywords: alcoholism; COVID-19 pandemic; Lesch typology; vaccination, liver damage; neurological damage.

1. Introduction

The COVID-19 disease was caused by the new coronavirus SARS-CoV-2 (Severe Acute Respiratory Syndrome Coronavirus-2), discovered in late 2019 in China, where the infection first occurred. Soon after it developed its pandemic potential with more than 100 million people being infected and 2 million died worldwide. It ranges from asymptomatic to sever, even lethal multisystem forms [1, 2]. However, in addition to physical, the

Formatted: Font: Not Bold

Formatted: Font: Not Bold

Formatted: Font: Not Bold

Formatted: Font: (Default) Times New Roman, 18 pt, Bold

Formatted: Heading 1, Indent: Left: 0", Space Before: 0 pt, After: 0 pt, Line spacing: single

COVID-19 pandemic also impacted people's mental health. It contributed to the increase of global social tension, anxiety and discomfort. There was an increase in the consumption of psychoactive substances, among which alcohol was the most common, as a way of self-help [3]. Killgore et al. have conducted a research to determine the relation between the lockdown and social isolation during the pandemic and the increased frequency of alcohol intake. They asked 5,931 individuals to complete the Alcohol Use Disorders Identification Test (AUDIT). Over a six-month period, the consumption of alcohol and likely dependence increased on a monthly basis for the individuals that were under stay-at-home restrictions compared to those who were not [4]. Due to the nature of their illness, psychiatric patients represent a vulnerable social group, and as such their reaction to the COVID-19 pandemic resulted in the worsening of their psychopathological symptoms, among which anxiety and depression were the most frequently recorded ones [3].

Treatment for alcoholism is a complex, long process which aims at establishing abstinence, behavioural changes and adopting healthy lifestyle habits. The success of treatment depends on the applied methods: with a conventional approach (pharmacotherapy and psychotherapy) the favourable outcome is achieved by one-fifth of patients, whereas with the systematic model by almost three-quarters of patients. However, there is no universal treatment plan which can be applied to all alcohol dependent patients. In order to plan a treatment, triage a patient and design an individual treatment plan, it is essential to have a systematised classification which can provide clear guidelines on different ways of treatment and optimal therapy goals [5].

The Lesch Typology is based on the data gathered from a longitudinal, prospective study. By observing the alcoholism development and progression in 436 patients, 4 types of alcohol dependent patients were identified based on the patients' drinking patterns and origin of alcohol craving:

I. the "allergy model" (craving caused by alcohol)

II. the "anxiety model" (craving caused by stress)

III. the "depressive model" (craving caused by mood)

IV. the "conditioning model" (craving caused by compulsion).

The Lesch Typology has proven to be exceptionally useful in everyday clinical practice, as it offers both the classification of alcohol dependent patients and additional information on therapeutic goals, as well as the most appropriate treatment approach to each subtype of patients [6].

In 2017, hence before the COVID-19 pandemic, Vejnovic et al. conducted a survey of 105 alcohol dependents at the Clinic for Psychiatry, Clinical Centre of Vojvodina. The patients were classified in one of the four types based on the Lesch Typology. The majority of patients belonged to type III (60% of the respondents), then to type II (21.9%), type IV (10.5%) and to type I (7.6%) [7].

Our paper aims to compare the sociodemographic and clinical characteristics of the patients admitted to hospital for alcoholism treatment a year after the COVID-19 pandemic to the aforementioned results of the 2017 study.

The working hypothesis of this paper is the rising number of type II and type III alcohol dependent patients (as identified by the Lesch Typology) in the post-COVID-19 pandemic period compared to the pre-pandemic period, as a likely consequence of the stress, fear, problems and adversities that were caused by the pandemic, especially the change of routine, set patterns of living, fewer social contacts and constant uncertainty. Furthermore, we studied the protective effect of anti-COVID-19 vaccine on somatic complications in alcohol dependent patients and the length of their hospitalisation.

2. Materials and Methods

Formatted: Font: (Default) Times New Roman, 18 pt, Bold

Formatted: Heading 1, Indent: Left: 0", Space Before: 0 pt, After: 0 pt, Line spacing: single

The research was conducted as a retrospective cross-sectional study at the Addictions Unit of the Clinic for Psychiatry, Clinical Centre of Vojvodina in Novi Sad. It included 218 patients aged over 18 years who were diagnosed with alcohol dependence (ICD-10: F10.2) according to the ICD-10 criteria and who were admitted for treatment in the period from 3rd October 2022 to 31 October 2023, that is to say, after the last wave of the COVID-19 pandemic in Serbia in August 2022 [8]. All the patients included in the research gave their written consent to treatment and thus participation in this research when admitted to hospital. The patients with comorbidities, either psychiatric or somatic, were not excluded from the research. Furthermore, this is a comparative study as its findings are compared

to the findings of a similar study carried out at the Clinic for Psychiatry 6 years earlier, that is, before the pandemic.

The data was collected by viewing and reviewing electronic medical records using the Clinical Information System (CIS). The data mentioned were accessed for research purposes from 1st December 2023 till 21st December 2023 and none of the authors had access to information that could identify individual participants during or after data collection. –The following sociodemographic characteristics were used: gender, age, marital status and occupation, while the clinical characteristics included: the time period of alcohol consumption, the number of hospitalisations due to alcoholism, the duration of the last hospitalisation, lesions of the liver and nervous system, other psychiatric diagnoses, heredity to alcoholism, whether the patient had a COVID-19 infection as well as their COVID-19 vaccination status.

To classify the patients by the Lesch Typology, the MS Windows softer package for data processing available in public domain was used (www.lat-online.at). The obtained data was exported to Microsoft Excel and statistically processed. The IBM SPSS 27.0 software was used for descriptive and analytical processing of the data. The results were presented in tabular and graphical forms.

In order to determine the importance of vaccination against COVID-19 infection in the group of alcoholics, the length of the last hospitalization was used as the main characteristic. Vaccinated and non-vaccinated alcoholics were compared regarding the length of the last hospitalization. The length of hospitalization was taken as a marker

because it is comprehensive and implies that there were no serious complications during treatment and that the course of treatment and the outcome were favorable. Given that this is an observational study, the outcome of the hospital treatment was not detailed, but it was generated and presented only by the length of the treatment.

Formatted: Serbian (Latin, Serbia)

The paper was written in accordance to the Declaration of Helsinki on medical research conduct. The Ethical Board of the Clinical Centre of Vojvodina gave their approval for the conduct of the research on 1st December 2023, registered under number 00-281.

3. Results

Formatted: Font: (Default) Times New Roman, 18 pt, Bold

Formatted: Heading 1, Indent: Left: 0", Space Before: 0 pt, After: 0 pt, Line spacing: single

The research included 218 patients, of which 180 (82.6%) were male, and 38 (17.4%) were female (Figure 1).

Figure 1. Gender distribution of patients

The average age of the patients was 51 (SD±11) years. The youngest patient was 20, and the oldest was 77 years old. Based on their age, the patients were divided into several groups. The young adults group included patients ages 18 to 34, the group of the

early middle-aged adults comprised patients ages 35 to 50, middle-aged patients were 51 to 65 years old, and older adults over age 66. In our study, there were 87 (39.9%) middle-aged patients, 86 (39.5%) early middle-aged patients, 26 (11.93%) older adults and 19 (8.71%) young adults. The results are presented in a tabular form (Table 1).

Formatted: Indent: Left: 0", First line: 0"

Formatted: Indent: Left: 0"

Table 1. Patients' age range

Age group	Number of patients (n)	Percent (%)
**Middle-aged adults (51-65)**	87	39.9
**Young middle-aged adults (35-50)**	86	39.5
**Older adults (≥66)**	26	11.93
**Young adults (18-34)**	19	8.71
**Total**	218	100

Of the total number of patients, 79 (36.2%) were married, 75 (34.4%) were single, 58 (26.6%) were divorced, and 6 (2.8%) have lost their spouse/partner (Figure 2).

Formatted: Indent: First line: 0.5"

Figure 2. Patients' marital status

In relation to work status, 97 (44.5%) patients were unemployed, 94 (43.1%) were employed, and 27 (12.4%) were retired (Figure 3).

Formatted: Indent: Left: 1.81", First line: 0.5"

Figure 3. Patients' work status

Formatted: Indent: Left: 1.81", First line: 0.5"

The length of the average period of using alcoholic drinks was 20 (SD±11) years. The shortest period was 6 months, and the longest 50 years. The average number of hospitalisations of alcohol dependent persons was 2 (SD±1). The avreage duration of the last hospitalisation due to alcohol use disorder was 7 days (SD±5). The shortest hospitalisation lasted 1 day, and the longest 23 days.

Of the total number of patients, 161 (73.9%) did not have a positive family history of alcoholism, and 57 (26.1%) did (Figure 4).

Figure 4. Psychiatric heredity disposition to alcoholism

In relation to the Lesch Typology, 111 (50.9%) patients belonged to type III, 45 (20.6%) to type I, 37 (17.0%) to type II and 25 (11.5%) to type IV. The results are presented in a tabular form (Table 2).

Formatted: Indent: Left: 0", First line: 0"

Formatted: Indent: Left: 0", First line: 0"

Table 2. The Lesch Typology

Type by the Lesch Typology	Nubmer of patients (n)	Percent (%)
III	111	50.9
I	45	20.6
II	37	17.0
IV	25	11.5
Total	218	100

Of the total number of patients, 125 (57.3%) did not have liver damage, and 93 (42.7%) did (Figure 5).

Figure 5. Presence of liver damage

Of the total number of patients, 138 (63.3%) did not have neurological damage, and 80 (36.7%) did (Figure 6).

Figure 6. Presence of neurological damage

Formatted: Indent: First line: 0.35"

In addition to alcohol dependence, 117 (53.7%) patients had a psychiatric comorbid condition and/or symptom, and 101 (46.3%) did not. Out of the total of 176 psychiatric comorbid conditions and symptoms, 69 (39.2%) suffered from depression, 31 (17.6%) suicidality, 28 (15.9%) personality disorder, 23 (13.1%) anxiety, 22 (12 .5%) psychiatric disorders due to the use of psychoactive substances other than alcohol (heroin, marijuana,

opiates, cocaine, speed and ecstasy) and 3 (1.7%) psychotic disorders. The results are presented in a tabular form (Table 3).

Formatted: Indent: Left: 0", First line: 0"

Table 3. Psychiatric comorbid conditions and symptoms

**Psychiatric diagnosis**	**Number (n)**	**Percent (%)**
Depression	69	39.2
Suicidality	31	17.6
Personality disorder	28	15.9
Anxiety	23	13.1
Disorders due to the use of PS other than alcohol	22	12.5
Psychotic disorders	3	1.7
Total	176	100

146 (66.9%) patients had a COVID-19 infection, of which 79 (36.2%) were treated on an ambulatory basis, and 62 (30.7%) in hospital conditions. The other 72 patients (33.1%) did not suffer from COVID-19 infection. The results are shown graphically (Figure 7).

Formatted: Indent: First line: 0.5"

Figure 7. Presence of COVID-19 infection

In relation to the vaccination status against COVID-19 infection, 112 (51.4%) patients received the vaccine, and 106 (48.6%) did not (Figure 8).

Figure 8. Patients' status of vaccination against COVID-19 infection

The Mann-Whitney test shows that there is a statistically significant difference in the average length of the last hospitalisation in relation to the COVID-19 vaccination status ($p<0.05$).

4. Discussion

Formatted: Font: (Default) Times New Roman, 18 pt

Formatted: Heading 1, Indent: Left: 0", Space Before: 0 pt, After: 0 pt, Line spacing: single

Soon after its initial outbreak in China, the COVID-19 disease was declared a global pandemic by the WHO in March 2020 [9]. Alongside its great impact on people's physical health, social functioning and economics, this public health issue has a significant effect on their mental wellbeing, as well as on the life quality of each individual [10]. Restrictive measures such as isolation and quarantine, aimed at suppressing this contagious disease are the key to preventing the transmission of the virus, but they represent a psychological burden for a population, disrupting personal, family and social functioning. The consequences of the pandemic are significant among vulnerable groups such as psychiatric patients, who are on the social margin even in normal circumstances [11].

The change in day-to-day routine and set patterns of living imposed by the pandemic caused a sense of fear and anxiety among vulnerable individuals, thus increasing incidences of stress-related disorders, but also causing exacerbation of existing psychiatric disorders. Strict epidemiological control measures, acting as social stressors, may lead to serious mental disorders such as depression and/or anxiety among previously healthy

individuals or represent a huge burden for the mentally ill [11]. The appearance of fear, anxiety and depressive symptoms heightens the desire to consume psychoactive substances such as alcohol, as a form of self-help, especially during stressful periods [12].

In this study, a statistically significantly higher presence of alcohol dependence was observed in males. In the United States of America (USA), alcohol use disorder diagnosed more often in men (7%) than in women (4%) [13]. The incidence of alcohol dependence during the pandemic was higher among males in Canada, India, Iran and the USA [14]. The average age of patients dependent on alcohol was 51 years, with almost equal representation of young middle-aged and middle aged adults. The National Epidemiologic Survey on Alcohol and Related Conditions (NESARC) and the National Survey on Drug Use and Health (NSDUH) have similar results related to the age ranges of persons dependent on alcohol. The prevalence of alcohol use disorders decreases with age. The risk of developing alcohol dependence was higher among the respondents aged 30 to 54 years compared to those who were 55 years or older [15]. White et al. pointed to the increase in number of alcohol-related deaths in 2020 compared to 2019, with the highest mortality rate in the age group 55 to 64 years [16].

In relation to marital status, the majority of patients are married, a slightly smaller number are single, while a quarter are divorced. Research conducted in June 2021 showed that there was a lower chance of getting married, that is, a higher chance of divorce among the population of alcohol dependents [17]. In relation to their work status, the largest number of patients are unemployed, a slightly smaller number are employed, and the

fewest are retired. In their research on the use of psychoactive substances in Israel during the COVID-19 pandemic, Levy and colleagues concluded that the differences in work status were significant only among the respondents who used illegal drugs but not alcohol. The occurrence of equal representation of the employed and unemployed among the alcohol dependent persons is explained by the wide availability, popularity and social acceptability of alcohol beverages in Israel, which is also the case in Serbia [18].

On average, the number of hospitalisations of alcohol dependents is 2, and the average duration of the last hospitalisation is 7 days. Ahacic et al. have conducted a study which included 576 patients admitted to hospital for alcohol dependence. Nearly 50% of the patients had been hospitalised only once before, 34% three times or more, and 18% twice. Most patients were readmitted for treatment in the period between 2 and 4 years after the last hospitalisation [19]. Our study has found that almost three quarters of patients have a negative family history of alcohol dependence. Edenberg and colleagues point to the complexity of alcohol dependence as a genetic disease with variations in a large number of genes which increase the risk of the disease. Among the identified genes, the ones that stand out are ADH1B and ALDH2, which are included in the metabolism of alcohol and have the greatest impact on the manifestation of alcohol dependence. Twin studies in the USA and Europe suggest that the concordance for alcohol dependence ranges between 45% and 65% [20].

In relation to the Lesch Typology, 51% of the patients belonged to Type III, 21% to Type I, 17% to Type II and 11% to Type IV. In the period between 2006 and 2007, Penha

Zago-Gomes and Nakamura-Palacios have conducted research on alcohol dependent patients at the Universidade Federal do Espírito Santo hospital. Of 170 patients, 21.2% were classified as Type I, 29.4% as Type II, 28.8% as Type III, and 20.6% as Type IV. They concluded that, although different types of alcoholics have different clinical presentations, the age at first intake of alcohol and the length of withdrawal did not differ among the types according to Lesch's Typology [21]. Jakovljevic and colleagues compared clinical characteristics of alcohol dependents in Serbia and Austria in the period between 2011 and 2012. The sample of Serbian population showed a very high rate of anxiety disorders (89.8% compared to 26.5%), which was explained by traumatic historical events in Serbs. Suicidal tendencies, not related to alcohol use or withdrawal syndrome, are significantly higher in the Austrian population sample (1.6% compared to 13.2%). There was no difference between the two samples in Type IV patients (20.5% compared to 20.6%). The sample of our population had significantly more Type III patients (42.5% compared to 33.8%) [22]. When compared to the results of classification of alcohol dependents according to the Lesch Typology obtained by Vejnovic et al. in 2017, it is evident that there was no increase in type II or type III after the COVID-19 pandemic, hence we reject the working hypothesis. Almost the same number of Type IV and a slightly higher number of Type I dependents is observed in our study. A study on reaction of the population of 4 different countries (Lebanon, Portugal, Italy and Serbia) to the COVID-19 pandemic indicated that the respondents from Serbia had greater resistance to psychological difficulties and stress that the COVID-19 pandemic imposed with its epidemiological

measures than the other participating countries. The authors explain that the reason for this lies in specific sociocultural characteristics of Serbian population, such as previous experience with epidemics and the wars in Yugoslavia in the period between 1991 and 1999 [23]. We explain the increase of Type I, somatic and neurological comorbidities by the circumstances of the pandemic which led to difficulties in receiving medical assistance, non- treatment or inadequate treatment of non-COVID-19 diseases and conditions, given that the entire healthcare system was focused on the current pandemic. The COVID-19 pandemic has made significant disruption in health service delivery all around the world [24]. The unavailability of health care for non-COVID patients during the COVID-19 pandemic was caused both by the burden on the health system by a large number of COVID patients and by the overload and saturation of health workers who were mostly reassigned from their regular tasks to work in the COVID centers [25]. Takinig all mentioned together, it is clear why the COVID-19 pandemics has disrupted both preventive and curative services for communicable and noncommunicable diseases [25-27]. Many of essential services, like internal and neurological check-ups, diagnostic procedures, have been delayed by the healthcare facilities, [28-30] patients were also unable to attend follow-ups and acute care visits due to the fear and anxiety they experienced during the pandemic waves [31].

It was difficult for all categories of non-COVID patients to obtain adequate health care during the pandemic, and especially for vulnerable social groups such as the alcoholics. The pandemic itself and the changes caused by it in the health system led to an

increase in tension and uncertainty among the alcoholics, which contributed to a change in the way of drinking alcohol as a coping mechanisam, which usually ment an increase in the amount and frequency of drinking, and easily led to the appearance of alcohol-induced disorders such as delirium tremens or abstinence syndrome, which led to acute hospitalization [32]. These disorders are a main feature of the first type of alcohol addicts according to the Lesh typology. The restricted access to healthcare system during the pandemic may have influenced the patterns of alcohol dependence leading to the increase in somatic complication of alcocholisam and explaining our finding- the increase of Lesh Type I addicts in our sample. This discovery sheds new light on how external stressors, such as the unavailability of adequate health care, can lead to a change in the pattern of alcohol consumption.

Nearly 60% of patients have no lesions of the liver, that is, there is no elevation of the liver enzymes. In the study conducted in Kentucky, 69% of alcohol dependents admitted for treatment had elevated biomarkers (aspartate aminotransferase and cytokeratin 18) for liver damage, with women having more pronounced lesions compared to men [33~~24~~]. Neurological damage was present in 37% of patients. Meta-analysis results from 2018 indicated a prevalence of 46% of alcoholic dependents with peripheral neuropathy [34~~25~~].

In addition to alcohol dependence, most patients suffered from a psychiatric comorbid condition or symptom. Depression was the most prevalent with nearly 40%, followed by suicidality, personality disorder, anxiety and the use of psychoactive

substances other than alcohol with almost the same share, and there were only three patients with a psychotic disorder. Comorbid psychiatric disorders in patients with alcohol dependence are very common. On the one hand, alcohol dependence can induce the appearance of psychiatric disorders and on the other, disorders such as depression and anxiety can cause excessive intake of alcoholic beverages as a form of self-medication [3526]. Depressive disorders are most common among alcohol dependents, especially major depressive disorder with a prevalence of 33% as found in a 2019 study [3627].

67% of patients were infected with COVID-19. The number of patients treated in an outpatient setting was almost equal to the number of those who were treated in hospitals. 52% of patients received a vaccine against COVID-19. Bailey et al. compared the course of the COVID-19 disease among patients with and without alcohol dependence. They proved that, when infected with SARS-CoV-2 virus, alcohol dependant patients have a higher chance of being admitted to hospital compared to patients without alcohol dependence. Moreover, the course of the disease is more sever, the complications are more prominent and mortality rates higher [3728].

Literature data indicate the fact that patients with psychiatric disorders fall into a vulnerable category and if infected with the COVID-19 virus, they suffer more serious forms of the disease. – In the population of psychiatric patients, the highest vaccination rate was among those with depression and anxiety disorders. On the other hand, patients suffering from psychotic disorders had the lowest rate of vaccination. There are several possible explanations as to why unvaccinated psychiatric patients had more severe forms

of the COVID-19 infection. It is thought that they often ignored somatic symptoms and had less contact with primary health care. Also, it is possible that doctors focused more on the mental problems of these patients, and neglected somatic difficulties [3829]. Findings from our study indicated a statistically significant difference in the average duration of the last hospitalisation compared to the vaccination status against COVID-19. The fully vaccinated patients were hospitalised for alcoholism for a shorter period and the course of the treatment was without serious complications. Studies on COVID-19 vaccination in the period between January and May 2022 have shown high efficacy of all available vaccines in the period [390]. The vaccination led to a smaller number of critically ill patients requiring treatment in intensive care units and a lower mortality rate [4031].

There are some deficiencies and possible limitations of this study. First, the design of the research, it self, as an observational study of a retrospective nature does not provide the ability to determin the causality between the examined phenomena. To overcome this problem and to better understand casual relationships, a longitudinal study is needed to validate our results and make them generalizable. Second, the sample size may not be sufficient to account for the sample representativness. It is necessary to conduct a similar study in cooperation with several different psychiatric institutions throughout Serbia and region to shed a new light on the the complexities of alcohol abuse during crises and draw validated conclusions.

5. Conclusions

Compared to the pre-pandemic findings of alcohol dependents classification according to the Lesch Typology, there was no increase in types II and III after the COVID-19 pandemic. This might be explained by specific sociocultural characteristics of Serbian population and their higher tolerance of difficulties and stress imposed by the pandemic. However, we explain that the rise of type I alcohol dependents, with somatic and neurological comorbidities, was caused by the circumstances of the pandemic that led to difficulties in healthcare delivery and poor treatment of non-COVID related illnesses and the reorientation of the healthcare system to the pandemic treatment. More than a half of patients in our study had COVID-19, half of whom had been vaccinated. We have proven a positive effect of vaccination on the length and course of hospitalisation, which indicates the importance of implementing a full and comprehensive vaccination programme for vulnerable psychiatric patients, who are often neglected due to stigma.

Supplementary Materials: None.

Author Contributions: Conceptualization, D.K. and J.A.; data curation, L.L. and D.S.; formal analysis, D.K., V.V. and M.C.; investigation, V.K. and V.B.; methodology, B.S., J.A. and M.A..; project administration, S.B. and D.R.; resources, D.R. and D.S.; software, S.B. and V.B..; supervision, V.K. and B.S.; validation, V.V. and M.C.B.; visualization, L.L. and P.S.; writing—original draft, M.C. and M.C.B.; writing—review and editing, M.A. and P.S. All authors have read and agreed to the published version of the manuscript.

Formatted: Font: (Default) Times New Roman, 18 pt, Bold

Formatted: Heading 1, Indent: Left: 0", Space Before: 0 pt, After: 0 pt, Line spacing: single

Funding: This research received no external funding.

~~**Institutional Review Board Statement:** The Ethical Board of the Clinical Centre of Vojvodina gave their approval for the conduct of the research on 1st December 2023, registered under number of protocol 00-281. The study was conducted in according to the Declaration of Helsinki from 1975 (revised in 2013) [32].~~

Informed Consent Statement: Written informed consent was obtained from all subjects involved in the study.

Data Availability Statement: The data that support the findings of this study are available from the corresponding authors (D.K. and J.A.) upon reasonable request.

~~**Acknowledgments:** On this occasion, the authors would like to wholeheartedly thank the employees of the Psychiatry Clinic of the University Clinical Center of Vojvodina in Novi Sad for their technical, moral and advisory support during the preparation of this work, as well as for the time and space allocated for the collection of data used in the preparation of this study.~~

Formatted

Conflicts of Interest: The authors declare no conflicts of interest.

References

Formatted: Font: 18 pt, Bold

Formatted: Heading 1, Space Before: 0 pt, After: 0 pt, Line spacing: single

1. Singhal T. A Review of Coronavirus Disease-2019 (COVID-19). Indian J Ped. 2020;87(4):281-6.
2. Teo I, Chay J, Cheung YB, Sung SC, Tewani KG, Yeo LF et al. Healthcare worker stress, anxiety and burnout during the COVID-19 pandemic in Singapore: A 6-month multi-centre prospective study. PLoS One. 2021;22;16(10):e0258866.

3. Kuljančić D, Cvjetković Bošnjak M, Vejnović AM, Bjelan S. Pandemija SARS-CoV-2 virusa i njen uticaj na mentalno zdravlje psihijatrijskih pacijenata u odnosu na psihički zdrave osobe. Timočki medicinski glasnik. 2021;46(4):161-8.
4. Killgore WDS, Cloonan SA, Taylor EC, Lucas DA, Dailey NS. Alcohol dependence during COVID-19 lockdowns. Psychiatry Res. 2021;296:113676.
5. Patel AK, Balasanova AA. Treatment of Alcohol Use Disorder. JAMA. 2021;325(6):596.
6. Schlaff G, Walter H, Lesch OM. The Lesch alcoholism typology - psychiatric and psychosocial treatment approaches. Ann Gastroenterol. 2011;24(2):89-97.
7. Vejnovic AM, Dickov A, Kneževic V, Ratković D, Kolundžija K, Ivetić O. Karakteristike osoba zavisnih od alkohola u odnosu na Lesh-ovu tipologiju. U: Burgić Radmanović M, Račetović G, urednici. IV Kongres psihijatara BiH; 2017 Nov 16-19; Banja Luka, BiH; p. 210.
8. Batut.org.rs [homepage on the Internet]. Godišnji izveštaj o zaraznim bolestima u Republici Srbiji za 2022. godinu. 2023 [updated 2023 August 19; cited 2024 February 13]. Available from: https://www.batut.org.rs/index.php?content=2671.
9. WHO Director-General's opening remarks at the media briefing on COVID-19 - 11 March 2020 [Internet]. Available from: https://www.who.int/director- general/ speeches/detail/who-director-general-s-opening-remarks-atthe-media-briefing-on-covid- 19 11-march-2020.
10. Marković I, Nikolovski S, Milojević S, Živković D, Knežević S, Mitrović A et al. Uticaj poverenja javnosti i medija na nivoe anksioznosti i depresije među stručnim radnicima tokom COVID-19 epidemije u Srbiji. Vojnosanit Pregl. 2020;77(11):1201-9.

11. Kuljancic D, Cvjetkovic Bosnjak M, Siladji D, Hinic D, Veskovic D, Janjic N et al. The Role of COVID-19-Associated Fear, Stress and Level of Social Support in Development of Suicidality in Patients Diagnosed with Affective and Stress-Induced Psychiatric Disorders during the COVID-19 Pandemic-A Comparative Analysis. Brain Sci. 2023;13(5):812.
12. Roberts A, Rogers J, Mason R, Siriwardena AN, Hogue T, Whitley GA et al. Alcohol and other substance use during the COVID-19 pandemic: A systematic review. Drug Alcohol Depend. 2021;229(Pt A):109150.
13. White AM. Gender Differences in the Epidemiology of Alcohol Use and Related Harms in the United States. Alcohol Res. 2020;40(2):01.
14. Sarfraz A, Sarfraz Z, Sarfraz M, Thevuthasan S, Khan T, Inam I et al. The Incidence of Alcohol Use Disorders during the COVID-19 Pandemic. Psychiatr Danub. 2022;34(3):535-43.
15. Delker E, Brown Q, Hasin DS. Alcohol Consumption in Demographic Subpopulations: An Epidemiologic Overview. Alcohol Res. 2016;38(1):7-15.
16. White AM, Castle IP, Powell PA, Hingson RW, Koob GF. Alcohol-Related Deaths During the COVID-19 Pandemic. JAMA. 2022;327(17):1704-6.
17. Thomas NS, Kuo SI, Aliev F, McCutcheon VV, Meyers JM, Chan G et al. Alcohol use disorder, psychiatric comorbidities, marriage and divorce in a high-risk sample. Psychol Addict Behav. 2022;36(4):364-74.
18. Levy I, Cohen-Louck K, Bonny-Noach H. Gender, employment, and continuous pandemic as predictors of alcohol and drug consumption during the COVID-19. Drug Alcohol Depend. 2021;228:109029.
19. Ahacic K, Kennison RF, Kåreholt I. Alcohol abstinence, non-hazardous use and hazardous use a decade after alcohol-related hospitalization: registry data linked to population-based representative postal surveys. BMC Public Health 2014;14:874.

20. Edenberg HJ, Foroud T. Genetics and alcoholism. Nat Rev Gastroenterol Hepatol. 2013; 10(8): 487-94.
21. Penha Zago-Gomes M , Miyuki Nakamura-Palacios E. Tipologia de Lesch em alcoolistas no Brasil. J Bras Psiquiatr. 2009;58(3):169-74.
22. Jakovljevic M, Riegler A, Jovanovic M, Djordjevic N, Patek K, Lesch O et al. Serbian and Austrian Alcohol-Dependent Patients: A Comparison of Two Samples Regarding Therapeutically Relevant Clinical Features. Alcohol and Alcoholism. 2013;48(4):505- 8.
23. Öcal A, Cvetković V, Baytiyeh H, Santos Tedim FM, Zečević M. Public reactions to the disaster COVID-19: a comparative study in Italy, Lebanon, Portugal and Serbia. Geomatics, natural hazards and risk. 2020;11(1):1864-85.
24. Menendez C, Gonzalez R, Donnay F, Leke RGF. Avoiding indirect effects of COVID-19 on maternal and child health. Lancet Glob Health. 2020;8:e863–e864. doi: 10.1016/S2214-109X(20)30239-4.
25. Haileamlak A. The impact of COVID-19 on health and health systems. Ethiop J Health Sci. 2021 Nov;31(6):1073-1074. doi: 10.4314/ejhs.v31i6.1. PMID: 35392335; PMCID: PMC8968362.
26. WHO, author. Attacks on health care in the context of COVID-19. Geneva: WHO; [Jully 20, 2024]. https://www.who.int/newsroom/feature-stories/detail/attacks-on-healthcare-in-the-context-of-covid-19 .
27. WHO, author. COVID-19 significantly impacts health services for noncommunicable diseases. Geneva: WHO; [Jully 20, 2024]. https://www.who.int/newsroom/detail/01-06-2020-covid-19-significantly-impacts-health-services-for-noncommunicable-diseases .
28. Papautsky EL, Hamlish T. Patient-reported treatment delays in breast cancer care during the COVID-19 pandemic. Breast Cancer Res Trea. 2020;184(1):249–254. doi: 10.1007/s10549-020-05828-7.

29. Panagiotidis E. Nuclear medicine and oncology in the COVID-19 pandemic era. Hell J Nucl Med. 2020;23(Suppl):35–40.

30. Park C, Sugand K, Nathwani D, et al. Impact of the COVID-19 pandemic on orthopedic trauma workload in a London level 1 trauma center: the “golden month”. Acta Orthop. 2020;91(5):556–561. doi: 10.1080/17453674.2020.1783621.

31. Passos L, Prazeres F. Impact on mental health due to COVID-19 pandemic: cross-sectional study in Portugal and Brazil. Int J Environ Res Public Health. 2020;17(18):6794. doi: 10.3390/ijerph17186794.

~~23.~~32. Shuey B, Halbisen A, Lakoma M, Zhang F, Argetsinger S, Williams EC, et al. High-Acuity Alcohol-Related Complications During the COVID-19 Pandemic. JAMA Health Forum. 2024;5(4):e240501. doi:10.1001/jamahealthforum.2024.0501

~~24.~~33. Kirpich IA, McClain CJ, Vatsalya V, Schwandt M, Phillips M, Falkner KC et al. Liver Injury and Endotoxemia in Male and Female Alcohol-Dependent Individuals 19 Admitted to an Alcohol Treatment Program. Alcohol Clin Exp Res. 2017;41(4):747- 57.

~~25.~~34. Julian T, Glascow N, Syeed R, Zis P. Alcohol-related peripheral neuropathy: a systematic review and meta-analysis. Journal of Neurology. 2018;266:2907–19.

~~26.~~35. Dervaux A, Laqueille X. Comorbidités psychiatriques de l’alcoolodépendance. Presse Med. 2018;47(6):575-85.

~~27.~~36. McHugh RK, Weiss RD. Alcohol Use Disorder and Depressive Disorders. Alcohol Res. 2019;40(1):arcr.v40.1.01.

~~28.~~37. Bailey KL, Sayles H, Campbel J, Khalid N, Anglim M, Ponce J et al. COVID-19 patients with documented alcohol use disorder or alcohol-related complications are more likely to be hospitalized and have higher all-cause mortality. Alcohol Clin Exp Res. 2022;46:1023–35.

~~29.~~38. Bosnjak MC, Kuljancic D, Vejnovic A~~-~~M, Hinic D, Knezevic V, Ratkovic D~~, Bosic V, Vasic V, Sakic B, Segan D~~, et al. Rates of Vaccination against COVID-19 in Psychiatric Outpatients. J. Pers. Med. 2024;14(7):748.

~~30.~~39. Chirico F, Teixeira da Silva JA, Tsigaris P, Sharun K. Safety & effectiveness of COVID-19 vaccines: A narrative review. Indian J Med Res. 2022 Jan;155(1):91-104.

~~31.~~ Gholinataj Jelodar M, Mirzaei S, Saghafi F, Rafieian S, Rezaei S, Saatchi A, Dehghani Avare Z, Dehghan Niri M. Impact of vaccination status on clinical outcomes of hospitalized COVID-19 patients. BMC Infect Dis. 2024 Feb 23;24(1):254.

~~32. World Medical Association. World Medical Association Declaration of Helsinki: Ethical Principles for Medical Research Involving Human Subjects. *JAMA* 2013, *310*, 2191–2194.~~

40.

Formatted: Normal, Don't add space between paragraphs of the same style, Numbered + Level: 1 + Numbering Style: 1, 2, 3, … + Start at: 1 + Alignment: Left + Aligned at: 0.55" + Indent at: 0.8"

Dear Academic Editor and Reviewers,

First of all, I would like to express my gratitude for your positive assessment of my scientific work. We are aware that there are certain shortcomings in our article and we are grateful for your comments, which we hope have significantly contributed to improving the quality and scientific value of our article.

I will now present our responses to your objections and suggestions. I hope we were able to respond to your requests and thereby improve the quality of the work enough to be eligible for publication.

Respons to the reviewer 1 comments:

1. A section to the discussion focused on “Pandemic Healthcare Disruptions and the Rise of Type I Alcochol Dependence” has been added to the discusion chapter and the matter has been extesively explained citing new literature sources. I hope we met your request.
2. The methodology for how the effect of the Impact of Vaccination on Hospitalization Outcomes in our sample was measured has been explained in more detail in the Metodology section. I hope you will find it satissfactory.
3. A more detailed "Limitations and Future Directions" section has been added to the conclusion addresing both reviewer's comments. I hope it will be enough.

Respons to The Journal Requirements:

1. I hope my revised manuscript now meets the PLOS ONE's style requirements, including those for file naming. I included the tamplates you have suggested.
2. There are ethical and legal restrictions on sharing a de-identified data set, because data contain potentially identifying or sensitive patient information taking into account that those are psychiatric patients with the addiction diagnosis. We are oblidged to protect the privacy of the patients participants because this matter is so sensitive and easiy can geopardise the patients privacy and exposure them to the public wich could be disasterous and is not according to the law. The Ethical Commitee of Clinical Centre of Vojvodina oblidged us to act like this. That is why we stated that the data will be shared upon the request to the corresponding authors. The request from the Ethical Commitee of our institution can be made on the phone No. +381214843484.
3. We stated in the availability form like this according to our‘s Ethical commitee decision on our data sharing protocol. The data can be shared only on request to the corresponding authors.
4. The ethics statement appear only in the Methods section of our manuscript.
5. A separate caption for each figure has been added in the manuscript.
6. Review of the reference list has been done to ensure that it is complete and correct.

Dear Editor and reviewers, I hope that with these minimal changes that we have made in our scientific work and according to your requests, it has now been improved and meets the criteria for publication.

I hope for a soon and positive answer.

Sincerely,

Dr. Dusan Kuljancic
