# Supplementary material for: Clinical profile of alcohol dependent paintents according to Lesch Typology one year after the Covid-19 pandemic-comparative study
Source: PLoS One. 2025 May 23;20(5):e0324851. doi: 10.1371/journal.pone.0324851 (PMC12101639; doi:10.1371/journal.pone.0324851)
Supplement: Attachment — Submitted filename: Response to Reviewers.docx [file pone.0324851.s003.docx]

Dear Academic Editor and Reviewers,

First of all, I would like to express my gratitude for your positive assessment of my scientific work. We are aware that there are certain shortcomings in our article and we are grateful for your comments, which we hope have significantly contributed to improving the quality and scientific value of our article.

I will now present our responses to your objections and suggestions. I hope we were able to respond to your requests and thereby improve the quality of the work enough to be eligible for publication.

Respons to the reviewer 1 comments:

1. A section to the discussion focused on “Pandemic Healthcare Disruptions and the Rise of Type I Alcochol Dependence” has been added to the discusion chapter and the matter has been extesively explained citing new literature sources. I hope we met your request.
2. The methodology for how the effect of the Impact of Vaccination on Hospitalization Outcomes in our sample was measured has been explained in more detail in the Metodology section. I hope you will find it satissfactory.
3. A more detailed "Limitations and Future Directions" section has been added to the conclusion addresing both reviewer՚s comments. I hope it will be enough.

Respons to The Journal Requirements:

1. I hope my revised manuscript now meets the PLOS ONE's style requirements, including those for file naming. I included the tamplates you have suggested.
2. There are ethical and legal restrictions on sharing a de-identified data set, because data contain potentially identifying or sensitive patient information taking into account that those are psychiatric patients with the addiction diagnosis. We are oblidged to protect the privacy of the patients participants because this matter is so sensitive and easiy can geopardise the patients privacy and exposure them to the public wich could be disasterous and is not according to the law. The Ethical Commitee of Clinical Centre of Vojvodina oblidged us to act like this. That is why we stated that the data will be shared upon the request to the corresponding authors. The request from the Ethical Commitee of our institution can be made on the phone No. +381214843484.
3. We stated in the availability form like this according to ourՙs Ethical commitee decision on our data sharing protocol. The data can be shared only on request to the corresponding authors.
4. The ethics statement appear only in the Methods section of our manuscript.
5. A separate caption for each figure has been added in the manuscript.
6. Review of the reference list has been done to ensure that it is complete and correct.

Dear Editor and reviewers, I hope that with these minimal changes that we have made in our scientific work and according to your requests, it has now been improved and meets the criteria for publication.

I hope for a soon and positive answer.

Sincerely,

Dr. Dusan Kuljancic
