# Supplementary material for: Clinical profile of alcohol dependent paintents according to Lesch Typology one year after the Covid-19 pandemic-comparative study
Source: PLoS One. 2025 May 23;20(5):e0324851. doi: 10.1371/journal.pone.0324851 (PMC12101639; doi:10.1371/journal.pone.0324851)
Supplement: Attachment — Submitted filename: Response to Reviewers 2.docx [file pone.0324851.s004.docx]

Dear Academic Editor and Reviewers,

First of all, I would like to express my gratitude for your positive assessment of my scientific work. We are aware that there are certain shortcomings in our article and we are grateful for your new comments, which we hope have significantly contributed to improving the quality and scientific value of our article.

I will now present our responses to your objections and suggestions. I hope we were able to respond to your requests and thereby improve the quality of the work enough to be eligible for publication.

Respons to the reviewer 1 comments:

1. The Introduction section has been expanded with more detail on each one of the 4 Lesch types of alcohol addiction.
2. Furthermore, the the terminology you requested is changed throughout the text.

Respons to the reviewer 2 comments:

1. As for the explanation for the rejection of our hypothesis, it was indeed unsatisfactory. We have now offered several alternative explanations that together can explain the discovery we made.
2. All information we could find online about the Lesch typology software we used was included in the study. Other data on the metric characteristics of the software are not publicly available, at least to us. I hope this information we have provided will be satisfactory.
3. Regarding the quality of the language and the readability and comprehensibility of the text, we did our best. Hopefully the text is of better quality now.

Dear Editor and reviewers, I hope that with these minimal changes that we have made in our scientific work and according to your requests, will now be enough to improve sufficiently the manuscript and meet the criteria for publication.

I hope for a soon and positive answer.

Sincerely,

Dr. Dusan Kuljancic
